# Spontaneous rates exhibit high intra-individual stability across movements involving different biomechanical systems and cognitive demands

**DOI:** 10.1038/s41598-024-65788-6

**Published:** 2024-06-27

**Authors:** Ben H. Engler, Anna Zamm, Cecilie Møller

**Affiliations:** 1 Department of Psychology, Centre for Cognitive Neuroscience, Paris-Lodron-University of Salzburg, Salzburg, Austria; 2 Center for Music in the Brain, Department of Clinical Medicine, Aarhus University and The Royal Academy of Music Aarhus/Aalborg, Aarhus, Denmark; 3 Department of Linguistics, Cognitive Science and Semiotics, Aarhus University, Aarhus, Denmark

**Keywords:** Spontaneous rates, SMT, SPR, Natural frequencies, Musicianship, Internal tempo, Human behaviour, Cognitive neuroscience, Cognitive control, Perception, Motor control, Central pattern generators, Sensorimotor processing, Psychology

## Abstract

Spontaneous rhythmic movements are part of everyday life, e.g., in walking, clapping or music making. Humans perform such spontaneous motor actions at different rates that reflect specific biomechanical constraints of the effector system in use. However, there is some evidence for intra-individual consistency of specific spontaneous rates arguably resulting from common underlying processes. Additionally, individual and contextual factors such as musicianship and circadian rhythms have been suggested to influence spontaneous rates. This study investigated the relative contributions of these factors and provides a comprehensive picture of rates among different spontaneous motor behaviors, i.e., melody production, walking, clapping, tapping with and without sound production, the latter measured online before and in the lab. Participants (*n* = 60) exhibited high intra-individual stability across tasks. Task-related influences included faster tempi for spontaneous production rates of music and wider ranges of spontaneous motor tempi (SMT) and clapping rates compared to walking and music making rates. Moreover, musicians exhibited slower spontaneous rates across tasks, yet we found no influence of time of day on SMT as measured online in pre-lab sessions. Tapping behavior was similar in pre-lab and in-lab sessions, validating the use of online SMT assessments. Together, the prominent role of individual factors and high stability across domains support the idea that different spontaneous motor behaviors are influenced by common underlying processes.

## Introduction

Many human behaviors, such as walking, speaking, and playing music are inherently periodic. Researchers have developed different measures to capture the natural frequencies, that is, unprompted periodicities, pertaining to those behaviors. As performing a movement at its natural frequency is characterized, on a phenomenological level, by a feeling of ease and naturalness, these measures usually come in the form of spontaneous rates—rates at which subjects perform simple movements when asked to perform them at a pace that feels most natural or comfortable to them. Self-paced finger-tapping rate, for instance, commonly referred to as spontaneous motor tempo (SMT)^1^, has been measured in various contexts—ranging from clinical^2,3^ over developmental biological^4^ to comparative investigations^5^. SMT has shown consistent intra-individual stability across several trials of the same task^1,6,7^ (for an overview, see Ref.^8^), yet this measure is also being discussed to be subject to contextual influences like arousal and time-of-day^6,7^, to individual influences like musicianship and age^9–12^ and to interacting factors like chronotype^7^. Spontaneous production or performance rate (SPR) is a measure of the more cognitively complex act of producing musical sequences^13,14^—more seldomly speech sequences^15^. SPR has usually been assessed in musicians playing melodies on their instruments^13,16,17^. More recent studies have successfully employed tapping paradigms making it measurable in non-musicians^10,14^. SPR also shows stability within subjects and across different levels of motor complexity and different melodies^13,14^. The evidence for correlations between SMT and SPR, however, remains inconclusive^10,15^. Turning to periodic behaviors involving different biomechanical systems, walking has thus far not been investigated in the context of general (neuro-cognitive) psychology. Medical studies, however, have shown walking patterns to be different for clinical vs. normal populations^18^, yet generally stable within participants^19^, clustering around inter-step intervals of 500 ms independent of age, height or weight^20^. Clapping rates decrease with growing limbs and clapping consistency is higher in adults than in children^21,22^. Comparing rates across domains between subjects, one study found that spontaneous tempi did not differ between stepping on the spot and finger or toe tapping, though no within-subject correlational analyses were conducted^23^. Another study found significant correlations for tasks involving larger effector systems (galloping, crawling)^24^ Empirical evidence is thus clearly pointing towards a consistency of spontaneous rates concerning the same task. Within-subject assessments of spontaneous rates across different types of tasks, however, are rare and the role of individual factors like musicianship remains unclear^9–11^.

From a biological perspective, spontaneous rhythmic movements can arise from central pattern generating neural circuits controlled by the brain. These circuits, for instance found in the brainstem or spinal cord, exhibit stable periodicities once activated and can be theorized as internal oscillators^25,26^. Additionally, differences in spontaneous rates can be a result of specific biomechanical or anatomical constraints pertaining to the effector system in use, resulting in different states of minimal energy expenditure^27–29^. For example, the weight and length of a limb place rigid constraints on the speed at which it moves naturally^30^. Other theoretical approaches assume a cognitive internal time keeping mechanism^31,32^, sometimes theorized as an internal clock^33^. The extent to which spontaneous rates are determined by such an internal pacemaker, be it a clock or a central pattern generator, is not fully understood; and the extent to which this internal pacemaker is influenced by individual factors like musicianship is not either. Regardless of the underlying mechanism, spontaneous rates should be consistent across similar motor behaviors according to all theories. However, consistencies of spontaneous rates across behavioral domains of varying similarity have not yet been thoroughly investigated.

The overarching goal of this study was to provide a more comprehensive picture of the dynamics of spontaneous behaviors by assessing a yet unexplored conglomerate of behaviors within the same subjects. We employed eight different spontaneous rates measures: walking rate, clapping rate, SPR (playing two melodies using a tapping device), and four variations of the classic SMT task, i.e., finger-tapping with and without sound production, online pre-lab, and online in-lab. We sought to determine to what extent spontaneous rates are consistent within subjects across domains and to investigate differences due to biomechanical (walking, clapping, tapping), individual (musicianship), and contextual (in-lab, pre-lab, time-of-day) factors. The contextual comparison also served to validate the use of online SMT assessments which has become increasingly popular in recent years (e.g. see Ref.^11^ or Ref.^7^). We expected spontaneous rates to be generally associated with one another within participants, but exhibit pronounced differences due to different biomechanical and cognitive constraints. Varying magnitudes of associations between rates within participants should reflect these differences.

## Results

### Patterns and distributions of different spontaneous rates

**Figure 1:**
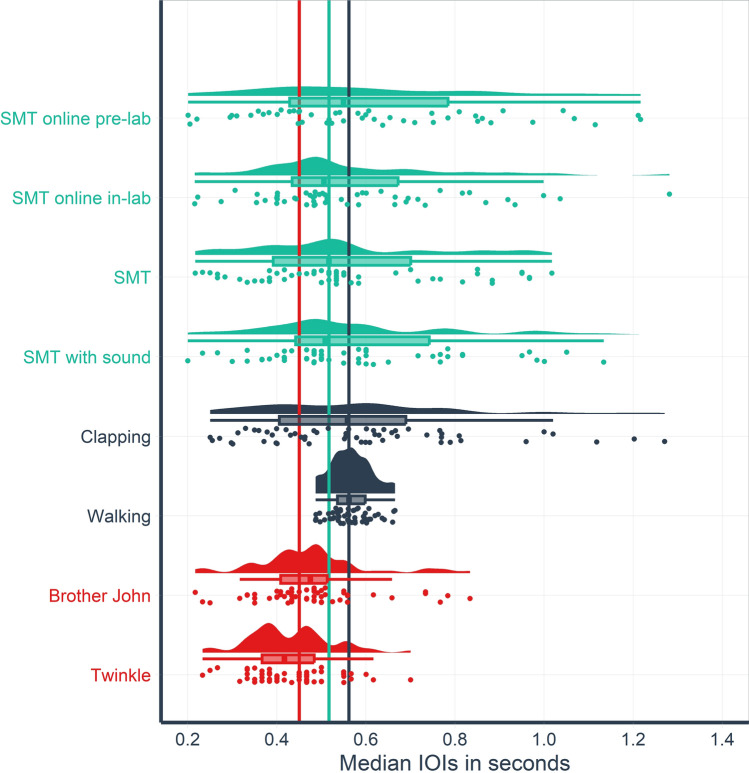
Raincloud plot (half density-plot, half dot-histogram) displaying distributions of spontaneous rates for different tasks. Dots display median inter-onset intervals (IOIs) for each subject and task. In case of several sessions per task (SMT online pre-lab and SMT with sound), a dot displays the median of single session medians. Boxplot crossbar indicates median, hinges represent 25th and 75th percentile. Boxplot outliers were removed because all single data points are displayed below it. Two data points lying beyond the upper x-axis limit were omitted for readability (for all ranges, see Supplementary Material S5). Vertical lines indicate group medians for variations of the spontaneous motor tempo (SMT) task (turquoise), clapping and walking (black) and music making tasks (red).

*Patterns in different spontaneous rates.* Figure 1 displays the distributions of spontaneous rates for all tasks. The distributions were vastly different for tasks of varying motor and cognitive complexity. The classic SMT tasks, as conducted in the lab, (Median *(Mdn)* = 0.52 s, Median absolute deviation *(MAD)* = 0.20 s) shares its main characteristics of a platykurtic, slightly positively skewed distribution with the clapping rates (*Mdn* = 0.55 s, *MAD* = 0.23 s) and the other slightly varied SMT tasks: SMT as measured several times (at least four; see methods section for details) using an online application before the lab visit (SMT online pre-lab; *Mdn*_*across*_ = 0.55 s, *Mdn(MAD*_*within*_*)* = 0.25 s; calculated from at least four sessions), SMT as measured using an online application in the lab (SMT online in-lab; *Mdn* = 0.51 s, *MAD* = 0.16 s), and SMT where every tap produced a sound (SMT with sound; *Mdn*_*across*_ = 0.51 s, *Mdn(MAD*_*within*_*)* = 0.14 s; calculated from three sessions). Involving the exact same effector system but different cognitive demands, the two SPR tasks reveal a much narrower range of tempi for the songs Twinkle, Twinkle, Little Star (henceforth referred to as Twinkle; *Mdn* = 0.42 s, *MAD* = 0.07 s) and Brother John (*Mdn* = 0.48 s, *MAD* = 0.09 s). Whereas the walking task exhibited the narrowest distribution (*Mdn* = 0.57 s, *MAD* = 0.05 s), it was centered around a value similar to all other tasks except SPR.

*SMT online pre-lab*. Generally, SMT online pre-lab session medians were lower on average (*Mdn* = 0.55 s, *MAD* = 0.25 s) than was reported in other large scale online studies, whereas the range (0.18–1.69 s) was similar (Ref.^11^: *Mdn* = 0.73 s; *M* = 0.78 s, *SD* = 0.33 s, *range*: 0.18–1.82 s; Ref.^7^: *M* = 0.65 s, *SD* = 0.25, *range*: 0.12–2.15 s). Across session MADs ranged from as little as 0.002 to 0.35 s, indicating a sample of very rigid to very flexible tappers.

### Associations between spontaneous rates

**Figure 2:**
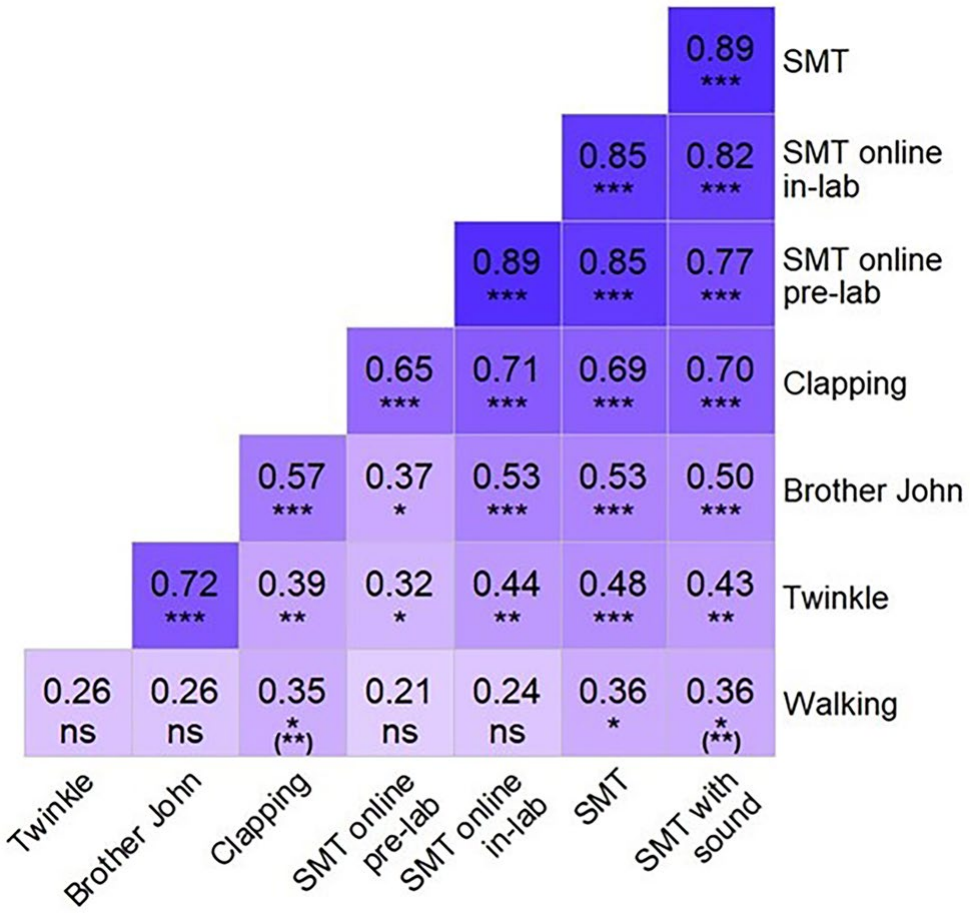
Heatmap displaying Spearman rank correlations between all spontaneous rates with FDR-corrected significance levels and uncorrected levels in parentheses. Significance level indicated by asterisks: ns = not significant, *p* >  = .05; * *p* < .05; ** *p* < .01; *** *p* < .001.

Spearman’s correlations were run for all 28 combinations of spontaneous rates measures. Additionally, the correlation between walking rate and leg length was calculated. Necessary *alpha* corrections for multiple comparisons were administered using the False Discovery Rate correction (*FDR,* Ref.^34^). All 28 correlations between spontaneous rates can be viewed in Fig. 2.

*Associations between spontaneous rates.* The analyses revealed strong correlations between clapping and tapping tempi in all variations of the SMT task (e.g., clapping and classic SMT: *r*(52) = 0.69, *p* < 0.001). Correlations with SPRs for both melodies (Brother John: *r*(53) = 0.57, *p* < 0.001; Twinkle: *r*(54) = 0.39, *p* = 0.005) as well as walking rate (*r*(52) = 0.35, *p* = 0.012) were smaller, but also significant. SPRs for the two melodies were highly correlated (*r*(53) = 0.72, *p* < 0.001). SPR for Brother John were also significantly correlated with all other rates (from SMT online pre-lab: *r*(45) = 0.37, *p* = 0.014) to clapping: *r*(53) = 0.57, *p* < 0.001), except walking (*r*(49) = 0.26, *p* = 0.076), and those for Twinkle only fell short of significance with walking rate (*r*(50) = 0.26, *p* = 0.070). In addition to the correlation with clapping mentioned above, walking rate was significantly correlated with SMT (*r*(48) = 0.36, *p* = 0.014) and SMT with sound (*r*(51) = 0.42, *p* = 0.012). A Spearman correlation revealed no significant correlation between leg length and walking rate (*r*(50) = 0.26, *p* = 0.704).

**Figure 3:**
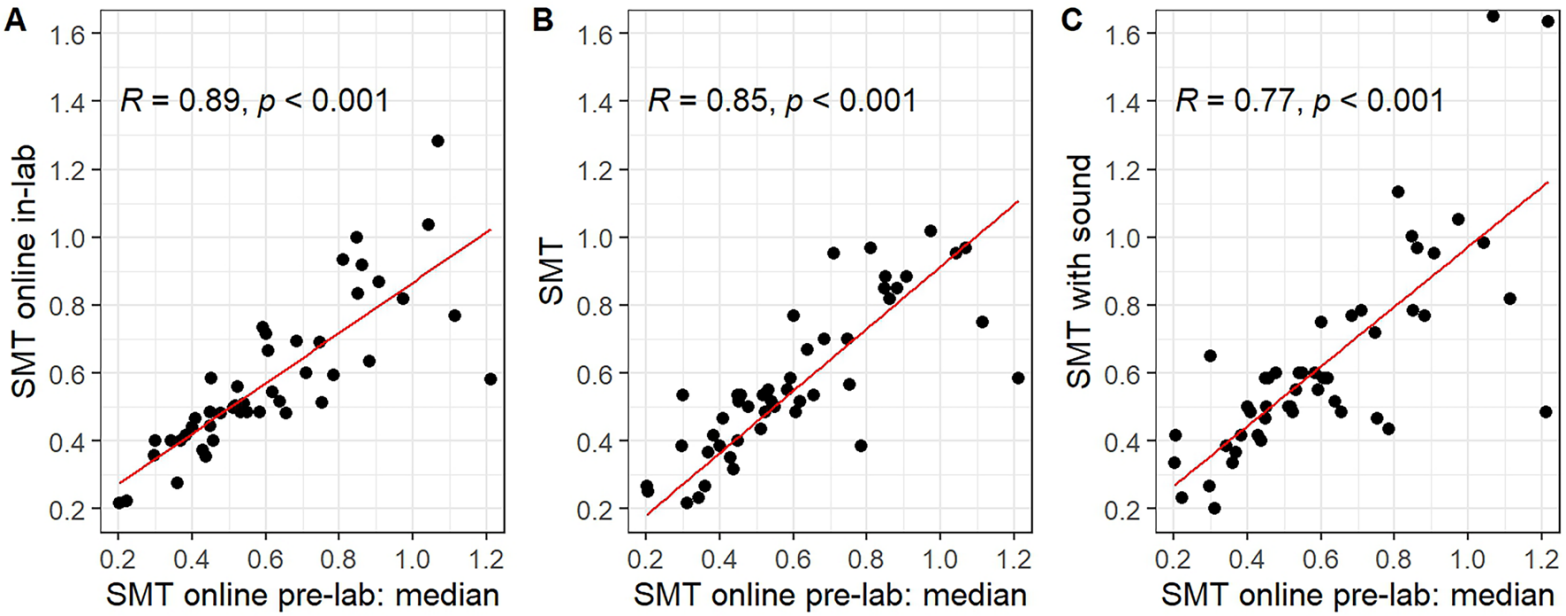
Scatterplots with red line indicating best linear fits. The medians of all median ITIs from the spontaneous motor tempo (SMT) online pre-lab sessions are plotted against the median ITIs for (**A**) SMT online (*n* = 47), (**B**) classic SMT (*n* = 48), and (**C**) SMT with sound (*n* = 50). All correlations were statistically significant (*p* < .001). X- and y-axes display IOIs in seconds.

*Validation of SMT online.* The plots in Fig. 3 show strong FDR corrected Spearman’s correlations between versions of the SMT task (pre-lab online, in-lab online, in-lab with sound). Participants were free to complete the online pre-lab sessions at their convenience, which could have led to an increase in variability due to a diverse range of factors. To assess whether variability was higher in pre-lab relative to in-lab SMT measurements, we conducted paired-samples *t*-tests on median absolute deviation divided by median tempo (MADM) of inter-tap intervals with SMT task (pre-lab online vs. in-lab online; pre-lab online vs. SMT) as factor. The *t*-tests indicated no significant differences between SMT online pre-lab and in-lab (*t*(45) = 1.04, *p* = 0.305) as well as SMT online pre-lab and SMT (*t*(47) = 0.69, *p* = 0.491) thereby not suggesting a difference in variability for the very same task conducted in- or outside the lab. We also compared MADMs between SMT online pre-lab assessments and the SMT with sound which turned out significant (*t*(48) = 3.74, *p* < 0.001). This likely does not point to differences due to in-lab vs. online assessments, but rather to differences due to different task demands.

### Effects of task and musicianship on spontaneous rates

A data-driven modelling approach was applied to investigate effects of task and musicianship on spontaneous rates. Models including *Task* (eight levels), including *Task* and *Musicianship* (musician/non-musician), as well as including *Task*, *Musicianship*, and the Task*Musicianship interaction as predictors were compared to each other and to a random intercept baseline model. As an alternative to the dichotomous musicianship variable, continuous estimates of Musical Training (MT), Perceptual Ability (PA), and General Musical Sophistication (G) obtained with the Goldsmiths Musical Sophistication Index^35^ were considered as well. The process resulted in a model using *Task* and *Musicianship* as fixed effects and participant as random effect. A detailed account of the modelling process can be found in the Supplementary Material (S1). The same modelling approach was administered using variability as Dependent Variable (DV). For reasons explained in the Methods section, no variability measures were calculated for walking and clapping rate, resulting in only six instead of eight levels for the factor task in this analysis.

**Figure 4:**
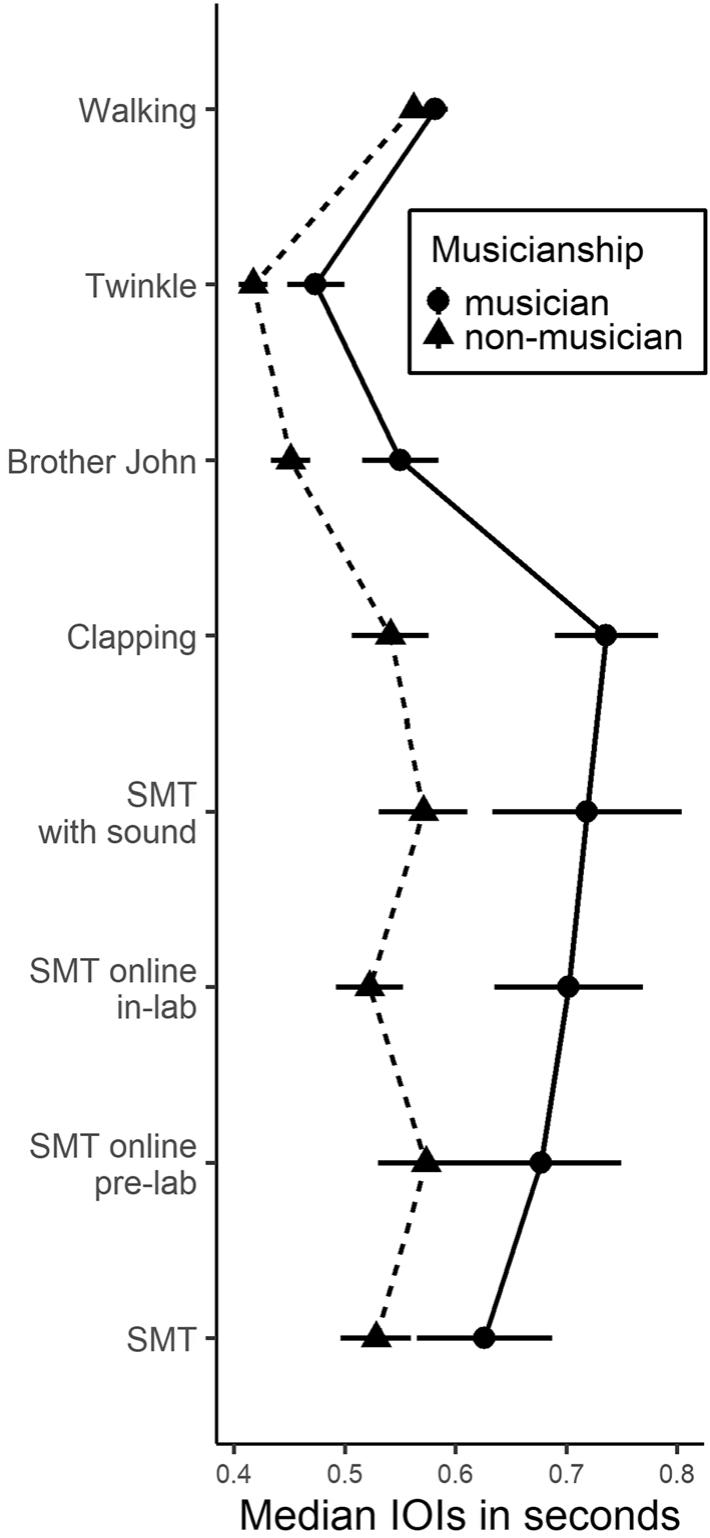
The effects of task and musicianship on spontaneous rates. Spontaneous rates in seconds compared for different tasks separately for musicians (dots) and non-musicians (triangles). Dots and triangles display means; error bars indicate standard errors of the mean.

*Tempo.* Task significantly predicted tempo (χ^2^(7) = 75.16,* p* < 0.001) and so did musicianship (χ^2^(1) = 5.60, *p* < 0.018). Averaged across tasks, musicians exhibited significantly slower tempi than non-musicians (*β* = − 0.11 [− 0.20; − 0.02]). In other words, musicians’ spontaneous rates were about 110 ms (+ /− 45 ms, standard error) slower than non- musicians’ (*t*(56.8) = 2.39,* p* = 0.021). Tukey-corrected analyses of estimated marginal means were conducted for the main effect of task. As shown in Fig. 4, tempi were different for different tasks. Averaged across both groups, participants’ spontaneous rates were significantly faster in the two SPR tasks than in all the others (see Supplementary Material S3 for all comparisons). As the cross-level interaction was not significant (χ^2^(7) = 11.90,* p* = 0.104), it was not included in the model. The visual depiction (Fig. 4) supports this decision as the two trajectories are quite similar. The intra-class correlation (ICC) calculated from the baseline model highlighted the importance of differences between participants (ICC = 0.49 [0.33; 0.63]). In the context of this analysis, a practical interpretation would be that if we were to inspect two random tasks within one participant, their correlation would be expected to lie between 0.33 and 0.63^36^. Of the alternative models including *MT*, *PA*, and *G* instead of the dichotomous factor *Musicianship*, only PA provided significant model predictions. In addition to task condition, PA significantly affected tempo (χ^2^(1) = 4.69, *p* = 0.030). The effect, however, was small, especially considering confidence intervals (*β* = 0.04 [0.004, 0.069]). The task*PA interaction was not significant and therefore not included in the model (χ^2^(7) = 13.10, *p* = 0.070). Exploratorily, we subsequently conducted a mediation analysis inspecting a potential mediating role of PA in the effect of musicianship on tempo. It resulted in no significant indirect effect (*β* = − 0.03, *p* = 0.282).

*Variability.* Inspecting the baseline model, between-subject differences were of smaller magnitude concerning tapping variability (ICC = 0.23 [0.05; 0.34]) than concerning tempo. Musicianship exhibited borderline significance in affecting tapping variability (χ^2^(1) = 3.84, *p* = 0.050). Across all tasks, musicians’ MADMs were lower than non-musicians’ (*β* = 0.02 [0.000001; 0.04]), meaning they tapped more consistently. The size of the effect, especially considering confidence intervals, is negligibly small. Task did not affect tapping variability (χ^2^(5) = 8.95, *p* = 0.111), and neither did any of the other independent variables (for a full account, see Supplementary Material S1).

### Effects of time of day on spontaneous rates

Using tempo (from single SMT online pre-lab sessions) as DV, two separate models included subjective and objective time of day as fixed effects, and participant as random effect (random intercept). The models used the same unconditional random intercept model as a baseline model. Two additional models had the same structure using within-session tapping variability as DV. There was no effect of subjective time of day (*β* = − 0.46 [− 4.21; 3.78]), nor objective time of day (*β* = − 0.58 [− 3.29; 4.05]) on tempo. Similar null results were found using variability as DV. For a full account, see Supplementary Material, S1.

## Discussion

In this study, we explored the dynamics of different spontaneous rates, focusing on intra-individual stability and task-dependent differences as well as the effects of time of day and music-related variables. We found robust intra-individual stability of spontaneous rates across different motor tasks (tapping, producing melodies, walking, and clapping), underscoring the enduring and powerful nature of internal factors driving spontaneous movements. Though strongly related, spontaneous rates exhibited nuanced differences related to the type of motor task performed. Averaged across all tasks, spontaneous rates were slower in musicians than non-musicians. These findings support the idea of a stable internal tempo^37,38^, simultaneously highlighting the intricate interplay between contextual (task-specific) demands and individual (musical) expertise. Additionally, we validated online SMT assessment by comparing the variability of our online pre-lab SMT assessment to the two in-lab assessments with identical task demands. Assessing SMT online gives researchers the opportunity to reach a wider audience of participants, make reliable assessments in exclusively online studies, and to save valuable in-lab time for more complex tasks, measuring SMT beforehand (or afterwards) using an online application. The SMT online pre-lab assessment did, however, yield more variable tapping than the SMT with sound. As the SMT with sound is characterized by auditory feedback for every tap, it is likely that participants were able to apply some continuous error correction regarding the consistency of their tapping. Furthermore, the finding that rates exhibited very high correlations not only when it comes to the exact same task on a different day (SMT online in-lab), but also for ones involving different equipment (SMT) as well as auditory feedback (SMT with sound), confirms and adds to prior findings of high stability over time within the classic SMT task^1,6^.

Our findings suggest a hierarchy of relatedness between different spontaneous movements that is due to specific cognitive and biomechanical similarities and differences reflected in the respective tasks. The SPRs for two melodies, for instance, were highly correlated with one another as well as moderately correlated with SMTs and clapping rates. The SPR tasks share a cognitive constraint that is absent in the other tasks: If familiar with the melody, there is only a certain range of tempi at which the production of that melody is recognizable. If played too fast or too slowly it loses its melodic characteristics. In tapping or clapping tasks in which there is no coherent pitch or rhythmic structure, only the temporal limits of beat perception and production constitute tempo boundaries. Accordingly, even though clapping rates exhibited significant correlations with all spontaneous rates, the ones with the SMT tasks were much more pronounced than with SPRs or walking rates. Analogously, walking, displaying a movements of greater motor complexity than the others, is only possible within a limited tempo range without performing a gait change (e.g., starting to run). As the walking task shares this biomechanical constraint with no other task, the correlations with other rates were smallest. Nonetheless, they were significant for clapping and two of the in-lab SMTs (SMT, SMT with sound), suggesting intra-individual stability even across very different domains of behavior. Thus, the aforementioned hierarchy of relatedness presents itself as follows: On the lowest level, all tasks share similar instructions, indicating the use of a shared internal mechanism to produce movement sequences. On the next level, the use of a common effector system in one subset of tasks (finger tapping for SMTs and SPRs as opposed to walking and clapping) indicates similarities due to biomechanical influences. These biomechanical influences, in turn, are overruled by the next level, namely the level of cognitive constraints. The presence of specific cognitive constraints is shared by one subset of tasks (two SPRs), whereas their lack constitutes another subset (SMTs, clapping). The SMT tasks without any sound (SMT online & SMT) were highly correlated with the SMT task with sound as they share the same effector system. Correlations with SPRs, however, though still using the same effector system, were substantially smaller.

The findings of this study can further be embedded into current discussions on the nature of internal oscillators as the underlying mechanism providing a preferred rate for auditory-motor actions and perception. Though our findings do not provide strong evidence for *any* internal time-keeping mechanism due to the vast differences in rates between tasks overall, they do provide evidence for a common underlying process in the form of an internal oscillator. The range of spontaneous rates was much larger for the tasks that had reduced cognitive and biomechanical constraints (SMTs and clapping) relative to the more complex tasks (e.g., SPR; for all ranges, see Supplementary Material S5). It follows that some people perform spontaneous tapping and clapping at rates faster or slower than realistically possible for walking and SPR, posing the question how they can be assumed to be influenced by a common underlying process? A recent study used a synchronization-continuation tapping task to assess people’s preferred motor rates and consistently found bimodal distributions^39^. As the underlying endogenous rhythm can be theorized as an internal oscillator^40^, it follows that the relation between the oscillator and its manifestations as a spontaneous rate can be described in terms of (sub-)harmonics. The relationship between an internal oscillator and a spontaneous rate can easily be 1:1 in one task, and 1:2 or 2:1 in another. Even ternary relationships are imaginable, though humans seem to generally prefer binary ones (see for a perceptual study in this context, Ref.^41^). This is also sensible from the perspective of music perception: It is generally agreed upon that a metric pattern’s highest level is always isochronous, and that, in cases where this does not hold, the level of subdivisions is^42^. Participants who exhibit a spontaneous rate at a harmonic tempo in relation to an assumed internal oscillator could clearly be mentally subdividing the pace of a self-sustaining internal oscillator. This notion would also be able to explain the rather few outliers we observed in the very high correlations between different SMT tasks. Anecdotal evidence from watching participants perform pilot SMT tasks further supports this idea, as some participants were observed to tap “in the air” between every tap on the respective surface. A future study could investigate this explanation by using an accelerometer to test for subdivisions in tapping tasks and delve into the reasons why some people might be more prone to subdividing than others. Taken together, our results and the ideas developed here support the idea of an internal oscillator as the underlying mechanism for spontaneous rates.

Though spontaneous rats are theorized to reflect properties of internal oscillators, they cannot provide the most accurate estimation of the theorized oscillator’s natural frequency itself, not least because of the possibility of subdividing. Thus, our results cannot provide direct evidence for an underlying internal oscillator. Estimating internal oscillator properties (first and foremost, eigenfrequency and flexibility) can be done in a more sophisticated way using synchronization-continuation tasks^12,39^. As those tasks have so far been mainly focused on tapping paradigms, it would be interesting to apply the method of synchronization-continuation tasks as a means of estimating internal oscillator properties to the range of tasks from this study. This could provide further evidence that an internal tempo indeed influences different types of motor behaviors in the way theorized by an internal oscillator model.

This study found that musicians exhibited overall slower spontaneous rates than non-musicians. This finding sheds some light on the hitherto little conclusive role that music-related variables may play in the production of spontaneous movements. Though it has been repeatedly shown that musicians exhibit enhanced temporal stability in tapping tasks^9,10,14^, the notion that they exert slower spontaneous movements remains controversial. It has been proposed that musicians possess a superior ability to track auditory-motor events over longer time scales, resulting in slower spontaneous rates^9,14^. Whereas one study has found this to be the case for SPRs^14^ and another one for SMTs^9^, there is also evidence to the contrary^10^. We found a significant main effect of musicianship but no interaction between musicianship and task, suggesting that musicianship did not influence different spontaneous rates to different degrees. Our findings furthermore tentatively support the explanation of superior event-tracking abilities in musicians. A mediation analysis including self-rated perceptual abilities as a potential mediator did not reveal a significant indirect effect. In the model including musicianship and perceptual abilities as predictors (as opposed to only musicianship), however, musicianship did no longer significantly predict tempo. This was not the case when inspecting a potential mediating role of musical training and is indicative of a lack of power with respect to the indirect effect. The role of perceptual abilities in spontaneous movements should thus be further investigated in a future study employing specific paradigms to assess participants’ event-tracking abilities. Additionally, future investigations could specifically recruit distinct musician groups (e.g., different instrument groups) to further disentangle what it is that makes musicians exhibit slower spontaneous rates.

Our findings from the SMT online pre-lab assessments challenge the idea of a straightforward effect of time of day—be it subjective, i.e., hours since waking, or objective—on the tempo and variability of SMT. Whereas some previous studies have found a small effect of speeding up throughout the day^11,16^, a more recent study has shown that this effect depends on an individual’s chronotype (morning type/lark vs. evening type/owl). SMT was found to speed up throughout the day for evening-types. For morning types the SMT was faster in the mornings, but did not change over the course of the day^7^. Thus, the fact that we did not assess chronotype and that the sample size was small in comparison to Ref.^7^ might have been critical for the lack of effect. Moreover, participants did not complete the online tasks at specific times but could do so at liberty. Though providing a more field-like setting, this might have further attenuated any potential effect. In sum, it seems to be the biological clock with preferences related to chronotype rather than one of time per se that influences spontaneous finger-tapping behavior. In conjunction with this study’s main finding of high intra-individual stability, this indicates that the role of individual factors and an internal tempo play a very prominent role concerning differences in spontaneous rates. The role of contextual factors like time of day, however, remains to be more directly addressed in future research, even though the data from this study does not provide evidence for it.

There are some limitations of this study that affect the generalizability of our results with respect to two aspects: the assessment of walking rates and the biomechanical characteristics we assessed. The only biomechanical characteristic we directly assessed was leg length. Future research should address the question of direct biomechanical influences in the context of spontaneous rates for other movements such as clapping as well. The assessment of walking rates lacks some ecological validity as it had to take place in a room of relatively short length. Though we tried to counter this in the extraction process (for a full account see Supplementary Material S6), some distortion of the data cannot be ruled out. A valid alternative would be to assess walking rates over a time span similar to the one we enforced in the clapping task.

In conclusion, tapping, melody production, walking, and clapping rates exhibited high consistency within participants and showed distinct differences related to cognitive and biomechanical constraints and to the individual factor musicianship. These findings support the idea that spontaneous rates in different motor tasks are influenced by common underlying processes, theorized as internal self-sustaining oscillations. As these oscillations ultimately represent very basic neural mechanisms, they provide a temporal frame of reference for perception and action in general. This means that estimating the role of contextual and individual factors influencing spontaneous rates is also of importance beyond the realms of un-cued spontaneous movements, as they may greatly impact an individual’s coordination with external events.

## Methods

### Participants

We recruited 60 participants aged 18–35 years with normal hearing and motor function and no neurological/psychiatric disorders. All data from one participant were excluded due to general difficulties following instructions. This left 59 participants (39 female, 5 self-reported left-handed) of 22 nationalities (mean age = 23.27 years, range = 18–35 years). Most participants grew up in Denmark (33.89%), followed by Poland (11.86%) and 16 other nations. According to the musician rank item (‘Which title best describes you?’) from Ollen’s Musical Sophistication Index (OMSI)^43^, the sample consisted of 14 nonmusicians and 30 music-loving nonmusicians (75% nonmusicians total), nine amateur musicians, one serious amateur musician, three semiprofessional musicians, and two professional musicians (25% musicians total). The sample’s average scores on *General Musical Sophistication* (*G; M* = 64.20, *SD* = 22.20, *range:* 28–118), *Musical Training* (*MT; M* = 18.88, *SD* = 11.87, *range:* 7–47), and *Perceptual Abilities* (*PA; M* = 42.20, *SD* = 10.72, range: 15–63) as assessed by Goldsmiths Musical Sophistication Index (Gold-MSI, Ref.^35^) were lower than in the large population studied in Ref.^35^ (M_G_ = 81.85 [20.62], M_MT_ = 26.52 [11.44], M_PA_ = 50.20 [7.86]).

The study protocol was approved by the Institutional Review Board at Aarhus University (approval no. 2023–007). Participants provided written consent according to the Declaration of Helsinki and received a cinema voucher as compensation for their time. The in-lab part of the study took place at the Cognition and Behavior Lab (COBE Lab) at Aarhus University, Denmark.

### Equipment and stimulus materials

Table 1 provides an overview of all tasks used for assessment of spontaneous rates. Online SMT assessments were done using a Shiny web application^44^ which was developed using the JavaScript library jsPsych^45^ embedded into psychTestR^46^. Participants were free to use any device with a touchscreen, clickpad, or mouse (no laptop touchpad or keyboard; see Supplementary Material S2 for a full account). While in the lab, participants completed the SMT online task once more sitting at a desk, but again using their own device. All other tasks were coded in Python using PsychoPy^47^ and executed locally on HP Desktop PCs. Sound was delivered via Creative Sound Blaster Sigma Tactic3D USB Headsets at a comfortable level which could be adjusted by the participants if necessary. The metal grid used to collect taps in the other tapping and melody production tasks, were attached to a Makey Makey (https://makeymakey.com/) that was connected to a Desktop PC via USB. Auditory stimuli used in the SMT with sound and SPR tasks were pure sine tones with a duration of 100 ms, including a 10 ms fade in and fade out. They were created using version 3.3.2 of Audacity®. In the SMT with sound task every tap on the metal grid produced a 523.25 Hz pure sine-tone. The series of tones produced by tapping in the SPR tasks formed two mostly isochronous melodies, chosen for their popularity: *Frère Jaques* (Brother John) and *Ah! vous dirai-je, Maman* (Twinkle, Twinkle, Little Star). Brother John consists of 20 quarter notes, 8 eighth notes, and 4 half notes, whereas Twinkle, Twinkle, Little Star consists of 36 quarter notes and 6 half notes. Both melodies were in C Major starting on C4 (261.63 Hz). Sounds produced when walking and clapping were recorded using a Zoom H2 audio recorder device. Questionnaires were administered using psyquest (https://github.com/fmhoeger/psyquest) and psychTestR^46^.

**Table 1:** Overview of tasks employed in this study. DV = dependent variable; ITI = inter-tap interval; IOI = inter-onset interval; MADM = median absolute deviation divided by the median; Mdn = median; SMT = spontaneous motor tempo; SPR = spontaneous production rate. Because there were several pre-lab SMT online sessions Mdn and MADM are calculated within and across sessions.

Task	Device	Instructions	DV	N
SMT online pre-lab	Smartphone/Laptop/ Tablet	*Using the index finger of your dominant hand, please tap at a steady rate that feels comfortable to you*	Mdn(ITI)_within_ Mdn(ITI)_across_ MADM(ITI)_within_ MADM(ITI)_across_	50
SMT online in-lab			Mdn(ITI) & MADM(ITI)	52
SMT	Metal plate			55
SMT with sound				58
SPR: Brother John		*Please play the song using the index finger of your dominant hand.* [two practice trials for each song]		56
SPR: Twinkle				57
Walking	Audio recorder	*Please walk at a steady rate that feels comfortable to you, put your hands on the wall, and walk back*	Mdn(IOI)	54
Clapping		*Please clap your hands together at a steady rate that feels comfortable to you*		58

### Design and procedure

Participants were asked to complete an SMT online assessment at least five times before coming to the lab for one hour on a scheduled day. During this hour participants completed two pitch discrimination experiments that were related to a different investigation. For reasons pertaining to this other investigation, participants completed the *SMT with sound* task first. The order or subsequent tasks was randomized. After the tasks, participants completed a short background questionnaire, assessing demographic variables, and the Goldsmith Musical Sophistication Index (Gold-MSI^35^; General, PA, MT). With the exception of the walking and clapping tasks, all task instructions were written on the screen and the experimenter left the room during the assessment. The instructions for all tasks were held as similar as possible (see Table 1). 

*SMT online assessments (pre-lab and in-lab).* Participants completed this task five times before coming to the lab and one more time while in the lab. On the Shiny app, participants started tapping upon the sound of a whistle and stopped when it sounded again (after 15 seconds). Each time they completed the task, participants were asked for their “subjective” time of day (hours since waking up) and entered a personal identifier.

*SMT with sound.* Sitting in front of desktop PCs, participants were instructed to put on the headphones and tap to produce a tone ten times in order to familiarize themselves with the tapping device. After this, they completed this task three times for reasons pertaining to a different investigation.

*SMT task.* Instructions were identical to the online assessments, only this time participants used the lab equipment.

*SPR.* Participants were given two trials explicitly for practicing, with the same instructions as in the main trial (see Table 1) each for Brother John and Twinkle, Twinkle, Little Star. To ensure a truly spontaneous production of the melodies in the main trial, participants did not listen to the melodies beforehand but the two practice trials allowed them to recognize the songs themselves. They were then asked to “play” the respective melody again for a third time in the main trial. The two melodies were presented in random order within the session.

*Walking and clapping.* Participants were asked to walk from one to the opposite side of the room (length: 7.8 m) at a steady rate that felt comfortable to them, touch the wall, and walk back. This was repeated once. For the clapping task, participants faced away from the experimenter and clapped their hands at a steady rate that felt comfortable to them for thirty seconds, until the experimenter asked them to stop.

### Data analysis

All data cleaning and statistical analyses were conducted using R^48^ and RStudio^49^. Analyses of linear mixed effects models were performed using the lme4 package^50^. Audio extractions for clapping and walking tasks were conducted using MATLAB version 9.13.0^51^ and version 1.8.2 of the MIRToolbox^52^.

#### Dependent and independent variables

*Dependent variables (DVs).* Table 1 provides an overview of DVs pertaining to the different tasks. For all tasks, tempo was calculated as the median inter-onset interval (IOI) within each session. For all tasks involving tapping, this is referred to as inter-tap interval (ITI). Relative tapping variability was calculated as the median absolute deviation (MAD) divided by the respective median (henceforth, this measure will be referred to as MADM). The MADM is a robust alternative to the coefficient of variation and preferable over other alternatives like IQR/median^53^. No measure of variability was considered for the non-tapping tasks (i.e., walking and clapping) for pre-processing reasons explained further below. For the SPR task, we assumed an influence of musical sophistication on relative tempo accuracy (half notes lasting twice as long as quarter notes etc.), meaning that non-musicians’ ITIs would be heavily skewed. To avoid this artefact, ITIs between half- and eighth notes were removed from the analysis. For Brother John, all taps from the eighth-note-series onwards were also removed. This left 12 ITIs for Brother John and 35 for Twinkle.

As there were several sessions for each participant in the SMT online pre-lab assessment (and not in the lab), we used participants’ *Mdn*_*across*_ in all analyses except the one investigating those single trials in particular checking for influences of time of day. *MAD*_*across*_ were not significantly different with respect to musicianship (*t*(18.93) = 0.28, *p* = 0.786), and there was no significant correlation with MT (*r*(47) = − 0.11, *p* = 0.471), PA (*r*(47) = 0.26, *p* = 0.072) or G (*r*(47) = 0.13, *p* = 0.379). This indicates that musicians in line with non-musicians vary in their tapping behavior across sessions, thus justifying the use of *Mdn*_*across*_ as a central tendency representative of a specific individual. Equally, the medians of the three session medians of the SMT sound task were used in all analyses.

*Independent variables.* Independent variables used in different analyses were task (SMT online pre-lab, SMT online in-lab, SMT, SMT with sound, SPR Brother John, SPR Twinkle, walking, clapping), *G*, *MT* and *PA* (as measured by the Gold-MSI), musicianship (musician/non-musician; assessed using the OMSI^43^ and then dichotomized), subjective time of day (in hours; measured using the question: “How many hours have passed since you woke up?”), objective time of day (in hours), and leg length (in cm; measured with shoes from floor to hip bone).

#### Pre-processing

Efforts were made to avoid exclusions on the participant-level, leveraging on the fact that all participants provided data for at least some tasks that were included in the analyses. Specifically, cleaning criteria were therefore applied at a session- and task-level for the SMT online pre-lab assessment (because each participant had several sessions), and at the session-level for all the other tasks. Individual IOIs were only considered in the non-tapping tasks (walking and clapping).

The only task-level inclusion criterion for the SMT online pre-lab assessment was that participants completed at least 4 sessions (after session-level pre-processing) in addition to the one they did in the lab. Otherwise, their SMT online pre-lab data was removed from all respective analyses.

The following session-level exclusion criteria were applied to all tasks except walking and clapping: (1) sessions with a MADM of 0.70 or higher were excluded, as this is a clear sign of non-isochronous tapping. (2) MADMs of 0.00 (indicating no tapping at all). (3) Single sessions were excluded if the median ITI was greater than 2000 ms or smaller than 200 ms. Those values are generally considered to reflect temporal limits of beat perception and production^54^. ITIs lying beyond those limits are therefore believed to be artefacts of mental subdividing and do not reflect spontaneous rates, but rather certain multiples or factors of those. Avoiding to treat those limits as arbitrarily strict, we considered absolute tapping variability (within-session MAD) before exclusion: If the session-median lie outside the limits, but session median + /− session-MAD did not, sessions were retained. Those three criteria will be referred to as cleaning criterion 1, 2, and 3, respectively.

The first two cleaning criteria were not applicable to the walking and clapping data for the following reasons: The audio extraction for walking and clapping data was mainly done using using the mirevents function from the MIRToolbox in Matlab^51,52^. In some cases, however, no automated onset extraction could be carried out. This was the case when peaks in the audio files did not correspond to onsets in the actual movement (e.g., a step). These cases had to be manually filtered out by comparing audible steps to the extracted onsets (for a full account of the extraction process, see Supplementary Material S6). As doing this manual comparison is not possible with regard to a measure of variability, we completely removed measures of variability for these two tasks. Furthermore, single IOIs below 150 and above 2100 ms (clapping), and below 330 and above 1100 (walking) were considered to be noise and thus excluded from single sessions. Though these might seem arbitrary, they were chosen upon careful auditory (no one clapped faster or slower) and visual (no one changed gait) observation of participants during data collection. If less than three IOIs remained, the session was excluded.

Considering the complexity of session exclusions, the number of participants included in the analyses varied, ranging from *n* = 46 (correlation between SMT online pre-lab and SMT online in-lab) to *n* = 57 (correlation between SMT with sound and clapping). For a full account, see Supplementary Material S4.

### Supplementary Information

Supplementary Information.

## Data Availability

The anonymized data collected in the context of this study are available from the corresponding author upon reasonable request.
