# Supplementary material for: Spontaneous rates exhibit high intra-individual stability across movements involving different biomechanical systems and cognitive demands
Source: Sci Rep. 2024 Jun 27;14:14876. doi: 10.1038/s41598-024-65788-6 (PMC11211469; doi:10.1038/s41598-024-65788-6)
Supplement: Supplementary file 1 — Supplementary Information. [file 41598_2024_65788_MOESM1_ESM.pdf]

Supplementary Material to:

Spontaneous rates exhibit high intra-individual stability across movements involving different biomechanical systems and cognitive demands

Ben H. Engler[1,2]*, Anna Zamm[3], and Cecilie Møller[2]

[1] Department of Psychology, Centre for Cognitive Neuroscience, Paris-Lodron-University of Salzburg, Austria

[2] Center for Music in the Brain, Department of Clinical Medicine, Aarhus University & The Royal Academy of Music Aarhus/Aalborg, Denmark

[3] Department of Linguistics, Cognitive Science and Semiotics, Aarhus University, Denmark

S1: Linear mixed effects models

Effect of task and musicianship on tempo and variability

Tempo. The final model included only task and musicianship as predictors, as both significantly affected tempo. As the explained variance increased substantially from the baseline model to the model with two predictors (change of $R^2_{residual}$ = .16; change of $R^2_{intercept}$ = .07) and performance measures were enhanced (see table below) compared to only using task, including them was interpreted as an improvement of the model. As the interaction effect was not significant ($\chi^2(7)$ = 11.90, p = .104), not affecting model predictions, and since there was no theoretical reason to assume an interaction effect either, it was not included in the final model. Lastly, we checked for an effect of leg length, which turned out not to be present in the data ($\chi^2(1)$ = 0.00, p = .990). As an alternative to the dichotomous variable musicianship, musical training, and general musical sophistication were included, but did not affect model predictions when adding them as an additional predictor to task (musical training: $\chi^2(1)$ = 2.72, p = .099; General: $\chi^2(1)$ = 2.73, p = .099). Perceptual abilities (PA), however, did affect model predictions ($\chi^2(1)$ = 4.69, p = .030). It seems to account for about the similar amount of residual and intercept variance as the model including musicianship ($R^2_{residual}$ = .18; $R^2_{intercept}$ = .08). The interaction between task and PA was not significant ($\chi^2(7)$ = 13.10, p = .070), not affecting model predictions.

Musicianship (The final model is underlined):

**Model**	**Parameters**	**AIC**	**BIC**	**Log likelihood**	**Deviance**	χ^2	***df***	***p***
Baseline (unconditional)	3	-296.51	-284.25	151.25	-302.51			
Task	10	-357.67	316.81	188.84	-377.67	75.16	7	<.001
Task + Musicianship	11	-361.28	-316.32	191.64	-383.28	5.60	1	.018
Task + Musicianship + interaction	18	-359.18	-285.62	197.59	-395.18	11.90	7	.104

Perceptual abilities (The final model is underlined):

**Model**	**Parameters**	**AIC**	**BIC**	**Log likelihood**	**Deviance**	χ^2	***df***	***p***
Baseline (unconditional)	3	-296.51	-284.25	151.25	-302.51			
Task	10	-357.67	316.81	188.84	-377.67	75.16	7	<.001
Task + PA	11	-360.37	-315.41	191.18	-382.37	4.69	1	.030
Task + PA + interaction	18	-359.47	-285.91	197.74	-395.47	13.10	7	.070

Variability. We did not include walking and clapping rates for reasons pertaining to the audio extraction process. The other 6 spontaneous rates were included. As neither task ($\chi^2(5) = 8.95$, $p = .111$), nor musical training ($\chi^2(1) = 0.91$, $p = .339$), general musical sophistication ($\chi^2(1) = 0.44$, $p = .505$) or perceptual abilities ($\chi^2(1) = 1.88$, $p = .170$) significantly affected model predictions, musicianship was the only predictor used in the final model, exhibiting borderline significance ($\chi^2(1) = 3.84$, $p = .500$).

Effect of time of day on tempo and variability in the SMT online pre-lab assessment:

Tempo. A vast amount of variance in the data turned out to stem from individual differences (ICC = 0.78 [0.61; 0.85]). There was no effect of subjective time of day ($\beta = -0.46$ [-4.21; 3.78]),

nor objective time of day (β = -0.58 [-3.29; 4.05]). Obviously, neither subjective ($\chi^2(1) = 0.03$, p = .864), nor objective time of day ($\chi^2(1) = 0.43$, p = .511) affected model predictions significantly as estimated using likelihood ratio tests. Including time as a polynomial (quadratic) term, yielded similar results (subjective time of day: $\chi^2(1) = 2.20$, p = .332; objective time of day: $\chi^2(1) = 1.13$, p = .569).

Variability. The two models concerned with tapping variability as DV again yielded similar null results. Generally, variability of within-session MADMs was substantial (M = 0.08, SD = 0.05). However, only a small proportion of this was due to individual differences (ICC = 0.23 [0.04; 0.37]). Other additional nested grouping variables were also unable to account for more variance, though ($ICC_{musician}$ = 0.02 [0.00; 0.17]; ICC_{sex} = 0.00 [0.00; 0.11]). As the effect of subjective time of day (β = -0.0006 [-0.002; 0.0005]) was just as absent as the one for objective time of day (β = -0.0004 [-0.002; 0.0006]), neither predictor improving model predictions (subjective: $\chi^2(1) = 1.07$, p = .301; objective: $\chi^2(1) = 0.69$, p = .405), a great part of the residual variance remained unexplained. Again, results were similar for time as a polynomial (quadratic) predictor (subjective: $\chi^2(1) = 1.79$, p = .409; objective: $\chi^2(1) = 1.23$, p = .540).

S2: Browser and device distribution

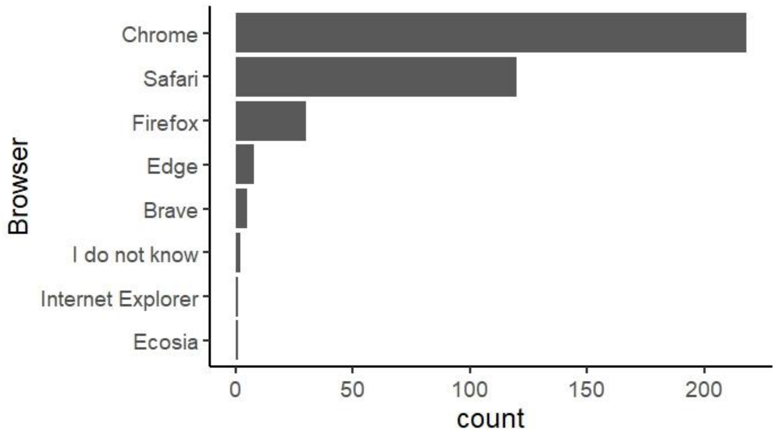

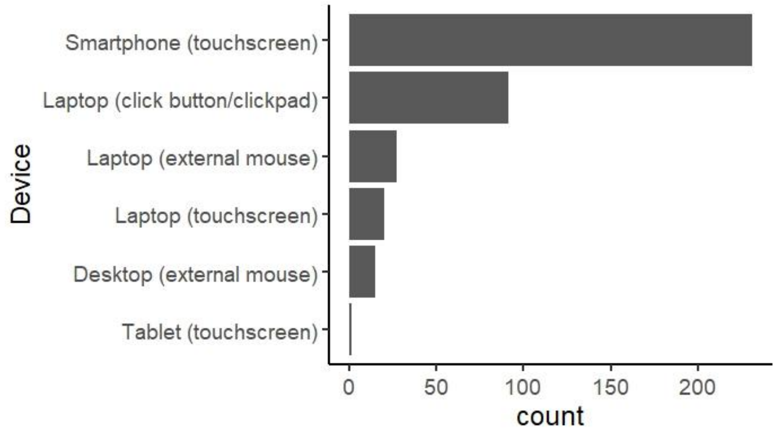

S3: Estimated marginal means comparisons for all levels of task (collapsed over levels of musicianship)

Comparison	*Estimate*	*SE*	*Df*	*t-value*	*p*
**Brother John - Clapping**	-0.11	0.03	374.64	-4.36	<.001
**Brother John - Twinkle**	0.05	0.03	374.38	1.83	0.600
**Brother John - walking**	-0.09	0.03	375.12	-3.33	.021
**Brother John – SMT online in-lab**	-0.09	0.03	375.69	-3.25	.027
**Brother John – SMT online pre-lab**	-0.12	0.03	375.61	-4.42	<.001
**Brother John - SMT**	-0.08	0.03	374.86	-3.06	.048
**Brother John – SMT with sound**	-0.13	0.03	374.75	-5.18	<.001
**Clapping - Twinkle**	0.16	0.03	374.50	6.23	<.001
**Clapping - Walking**	0.02	0.03	374.47	0.95	.980
**Clapping – SMT online in-lab**	0.03	0.03	374.99	0.98	.977
**Clapping – SMT online pre-lab**	-0.01	0.03	375.54	-0.24	1.000
**Clapping – SMT**	0.03	0.03	374.93	1.25	.916
**Clapping – SMT with sound**	-0.02	0.03	374.48	-0.82	.992
**Twinkle – Walking**	-0.13	0.03	374.97	-5.16	<.001
**Twinkle – SMT online in-lab**	-0.13	0.03	375.53	-5.06	<.001
**Twinkle – SMT online pre-lab**	-0.17	0.03	375.73	-6.20	<.001
**Twinkle – SMT**	-0.13	0.03	374.70	-4.90	<.001
**Twinkle – SMT with sound**	-0.18	0.03	374.61	-7.05	<.001
**Walking – SMT online in-lab**	0.00	0.03	375.53	0.04	1.000
**Walking – SMT online pre-lab**	-0.03	0.03	376.11	-1.14	.947
**Walking – SMT**	0.01	0.03	375.45	0.29	1.000
**Walking – SMT with sound**	-0.05	0.03	374.95	-1.76	.649
**SMT online in-lab – SMT online pre-lab**	-0.03	0.03	375.18	-1.18	.938
**SMT online in-lab – SMT**	0.01	0.03	375.14	0.24	1.000
**SMT online in-lab – SMT with sound**	-0.05	0.03	374.90	-1.78	.633
**SMT online pre-lab – SMT**	0.04	0.03	375.37	1.43	.841
**SMT online pre-lab – SMT with sound**	-0.01	0.03	375.08	-0.55	.999
**SMT – SMT with sound**	-0.05	0.03	374.49	-2.06	.441

S4: Pre-processing/Exclusion for different tasks

SMT online assessments: Pre-lab. No influences of browser or device could be detected in the data. Data was removed from analyses for six participants who completed the assessment less than four times. Additionally, three participants were removed due to cleaning criterion 3 and one participant misunderstood the task and provided no taps at all. This left data from a total of 50 participants.

SMT online assessment: In-lab. One participant did not provide any taps, six were removed due to cleaning criterion 1 and 3. This left data from a total of 52 participants.

SMT task. One participant was unable to perform the SMT task in the lab due to technical issues. Accommodating cleaning criterion 3, three more had to be removed, leaving a total of 55 participants.

SMT with sound. One participant was removed due to cleaning criterion 3; Otherwise, all data could be retained from this task, leaving data from 58 participants.

SPR. One participant experienced technical issues during both melodies. Two participants and one participant stated after the experiment that they were not familiar with the melody of Brother John and Twinkle, respectively. This left data from a total of 56 participants for Brother John and 57 for Twinkle.

Walking and clapping. One clapping audio file was lost due to technical issues. Otherwise, all clapping data could be retained, leaving data from 58 participants. After within-session cleaning, five participants' walking data had to be removed because of too much noise, leaving data from 54 participants.

S5: Ranges for all spontaneous rates

SMT online pre-lab: 0.20s - 1.22s

SMT online in-lab: 0.22s – 1.28s

SMT: 0.22sec – 1.02s

SMT with sound: 0.20s – 1.65s

Brother John: 0.22s – 0.83s

Twinkle, Twinkle, Little Star: 0.23s – 0.70s

Clapping: 0.25s – 1.27s

Walking: 0.49s – 0.67s

S6: Audio extraction process

First, the first author went through all audio files and spectrograms to assess time windows to retain in the subsequent analysis in Matlab. It was necessary to remove sections before and after the actual movements as they contained the experimenters' verbal instructions.

Further segments had to be removed for the walking data: In our walking task, participants walked from one end of a room to the other and back. This means that they accelerated and decelerated and stopped once. All these segments of clear acceleration and deceleration (at least the first and last step) and the stopping were thus removed from the audio files.

For the new, clean audio files, we used the mirevents function from the MIRToolbox in Matlab with the mirpeak contrast argument set to 0.15. This argument specifies how local maxima in the spectrogram, i.e., the peaks (in our case onsets: one clap or one step), are defined. A local maximum was thus defined as an onset (or peak) if the difference between it and surrounding (previous and successive) local minima was at least 0.15 times the total amplitude of the entire signal. Though this reliably extracted the peaks for most participants, there were some data files that were simply too noisy. Data files were considered unfit for further analysis if peaks from the audio files were calculated but they did not correspond to real event onsets in the form of claps or steps as evaluated by listening to the audio. An example illustrating this is one participant whose steps were masked by the sounds of the pants' fabric. Thus, no automated onset extraction could be carried out. Four such cases were excluded upon comparing extracted onsets and the respective medians to the audible steps.

We used an audio-based approach to extract walking rate due to the setting of the task. It was conducted in a 7.8m long lab room. Simply calculating steps/min or a similar measure would

thus not have been feasible. The advantage of this is that it allowed us to assess walking rate in a completely controlled setting. However, it also means that the task lacks some ecological validity because participants most likely accelerate and decelerate. An alternative would have been to assess walking rate just like we assessed clapping rates by letting participants walk freely in an unbounded space for a set amount of time. As popular gait analysis systems used in recent studies use similar or shorter walkway lengths[1], however, both options seemed viable.

[1] E.g., Al Jaja, A., Sue, T., Prenger, M., Seergobin, K. N., Grahn, J. A., & MacDonald, P. A. (2024). Alprazolam Reduces Freezing of Gait (FOG) and Improves FOG-Related Gait Deficiencies. *Parkinson's disease*, *2024*, 3447009. https://doi.org/10.1155/2024/3447009
